# A giant and extensive solitary Peutz–Jeghers-type polyp in the antrum of stomach: Case report

**DOI:** 10.1097/MD.0000000000008466

**Published:** 2017-12-08

**Authors:** Bai-Cang Zou, Feng-Fan Wang, Gang Zhao, Xiao-Lan Lu, Li Zhang, Ping Zhao, Hai-Tao Shi, Bin Qin, Xiao-Dan Guo, Jing Zhang

**Affiliations:** a Department of Gastroenterology, the Second Affiliated Hospital of Xi’an Jiaotong University, Xi’an, Shaanxi, China; b Department of Gastroenterology, Xi’an Children's Hospital, Xi’an, Shaanxi, China.

**Keywords:** case report, endoscopic submucosal dissection, endoscopic ultrasonography, hamartomatous polyp, peutz–jeghers-type polyp

## Abstract

**Rationale:**

A solitary Peutz–Jeghers-type polyp is a hamartomatous polyp which without either mucocutaneous pigmentation or a family history of Peutz–Jeghers syndrome (PJS). It can occur in all of the gastrointestinal tract, but it is extremely rare in the stomach.

**Patient concerns:**

A 53-year-old man was admitted to the local hospital with left upper abdominal pain lasting 2 weeks. A gastroscopy showed a giant and extensive bulging lesion on the greater curvature and posterior and anterior walls of the gastric antrum, involving three-quarters of the gastric wall. Endoscopic ultrasonography showed a muscularis mucosa lesion.

**Diagnoses:**

A solitary Peutz–Jeghers-type polyp in the antrum of stomach.

**Interventions:**

The patient underwent an endoscopic submucosal dissection (ESD).

**Outcomes:**

The patient recovered quickly, without any complications.

**Lessons:**

This is the second largest gastric solitary Peutz–Jeghers-polyp reported until now, and the largest gastric solitary Peutz–Jeghers type-polyp treated by endoscope.

## 1 Introduction

Peutz–Jeghers syndrome (PJS) is characterized by mucocutaneous pigmentation and hamartomatous polyposis in the gastrointestinal tract. If a patient has a hamartomatous polyp but no mucocutaneous pigmentation or a family history of PJS, he/she is diagnosed with a solitary Peutz–Jeghers-type polyp. A solitary Peutz–Jeghers-type polyp can occur in all of the gastrointestinal tract, but it is extremely rare in the stomach. Only 9 cases were reported. This study is aimed to report a case of solitary Peutz–Jeghers-type polyp in the antrum of stomach treated by endoscopic submucosal dissection (ESD). It was the second largest gastric solitary Peutz–Jeghers-type polyp reported till date and the largest gastric solitary Peutz–Jeghers-type polyp treated by endoscopy.

## 2 Case report

**Figure 1:**
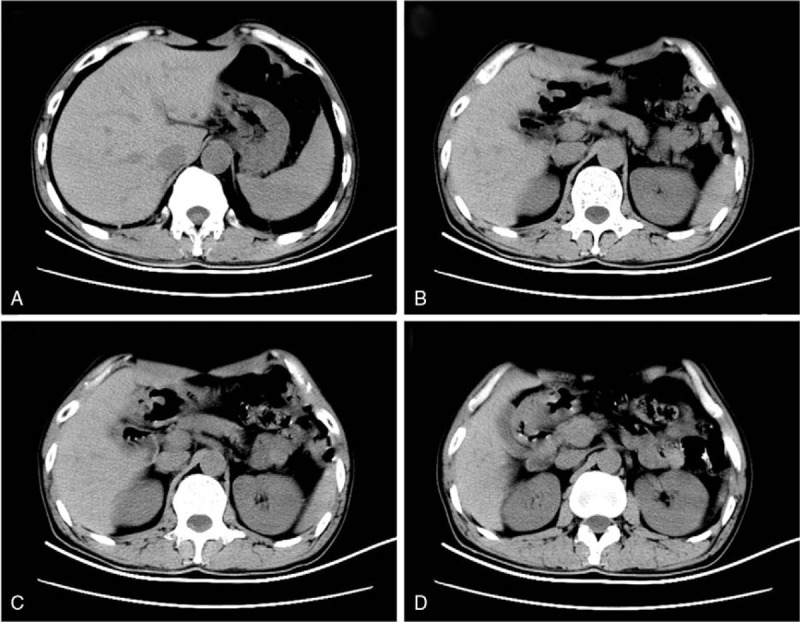
Abdominal computed tomography revealed a thickened gastric wall in the antrum.

**Figure 2:**
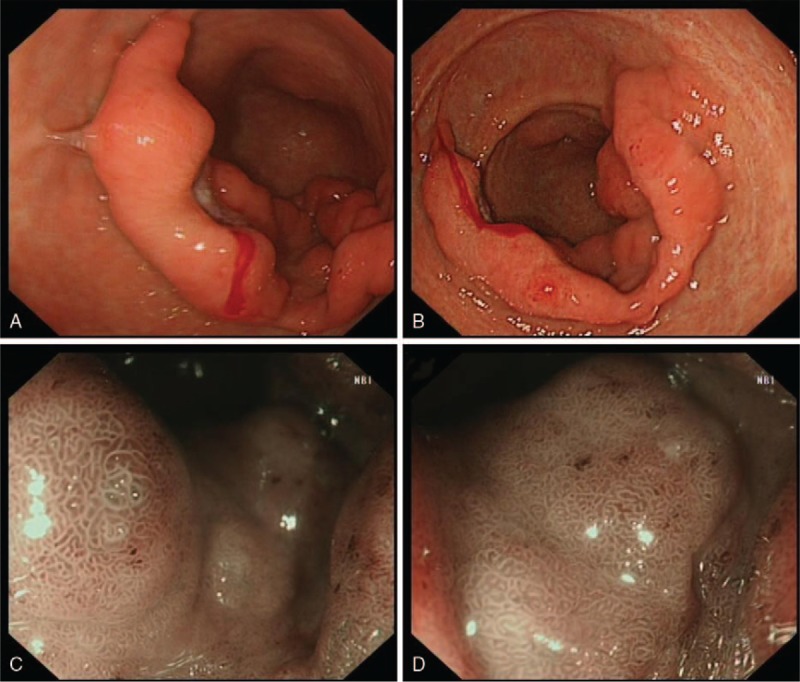
Gastroscopy showed a giant and extensive bulging gastric lesion located on the greater curvature and posterior and anterior walls of the gastric antrum, involving three-quarters of the gastric wall. The mucosal surface of the lesion was smooth without erosion or ulcer. Magnifying endoscopy with narrow-band imaging showed the almost normal microvascular morphology of the lesion.

**Figure 3:**
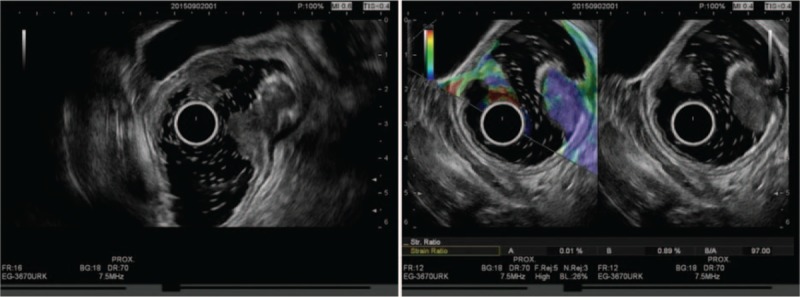
Endoscopic ultrasonography showed a low-echo area in the muscularis mucosae layer and no invasion into the submucosal layer, without enlarged celiac lymph nodes.

**Figure 4:**
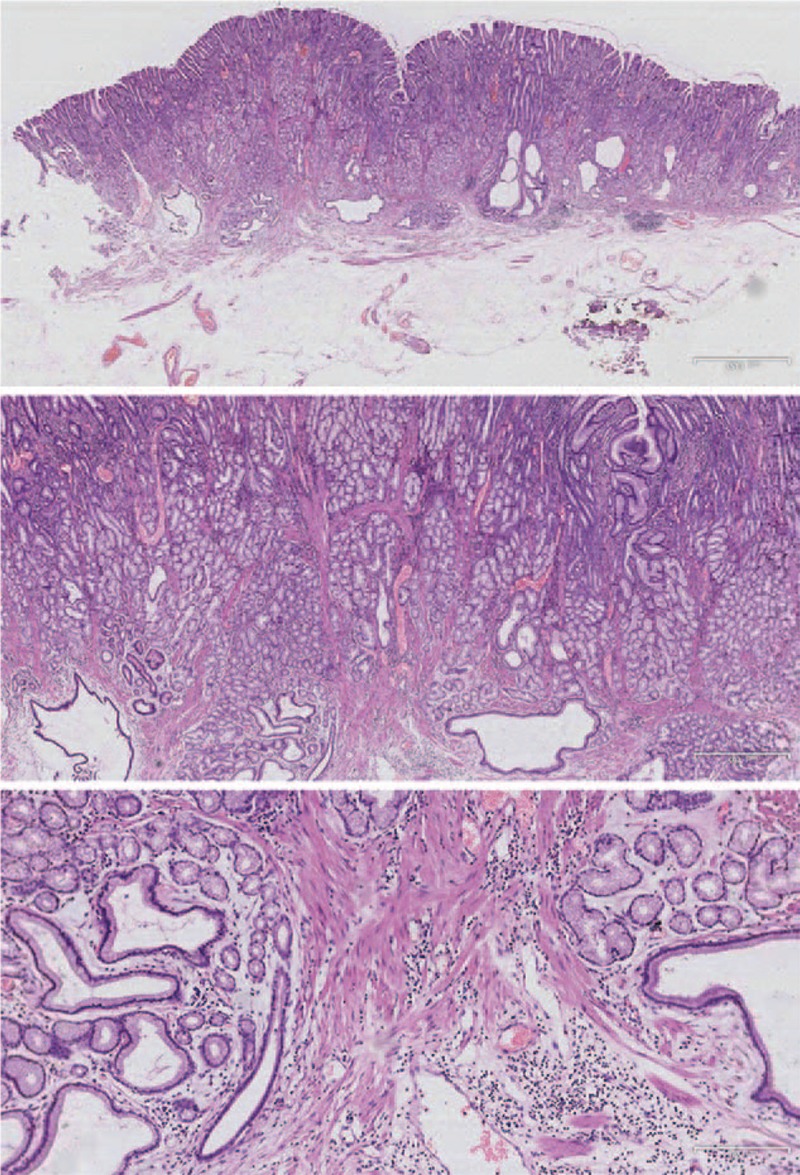
Histological examination of the endoscopic mucosal resection specimen showed that smooth muscle bundles from the muscularis mucosae extended to the polyp, forming a typical branch-like structure covering almost normal mucosa.

**Figure 5:**
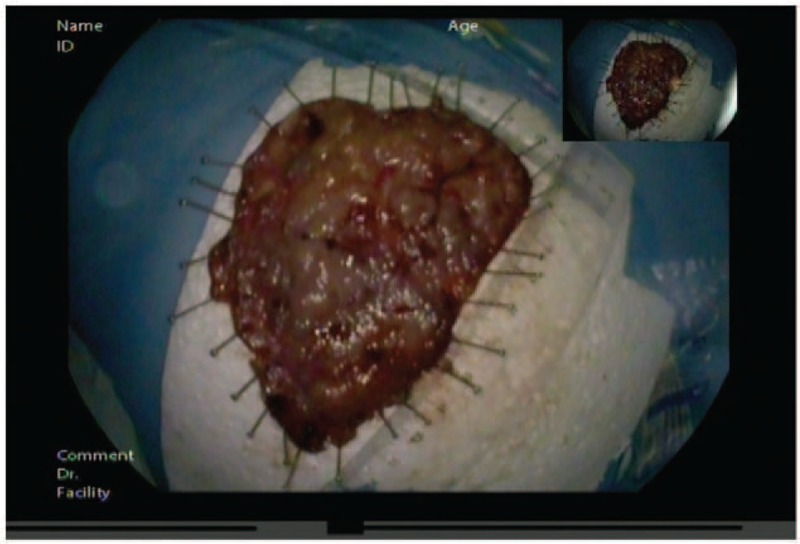
A lesion in the patient was treated by endoscopic submucosal dissection, and the large specimen measured in 110 × 80 × 4 mm^3^.

**Figure 6:**
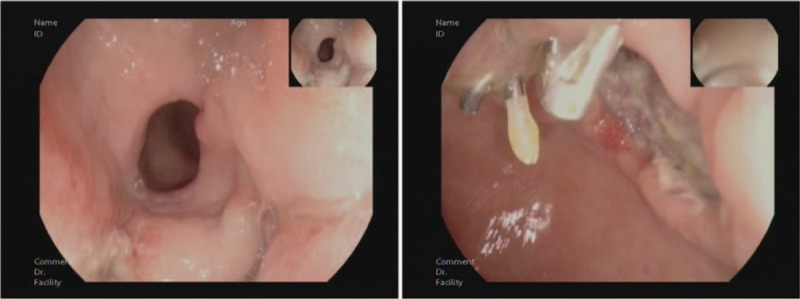
A gastroscopy on the fourth day after endoscopic surgery showed a large postoperative artificial ulcer.

**Figure 7:**
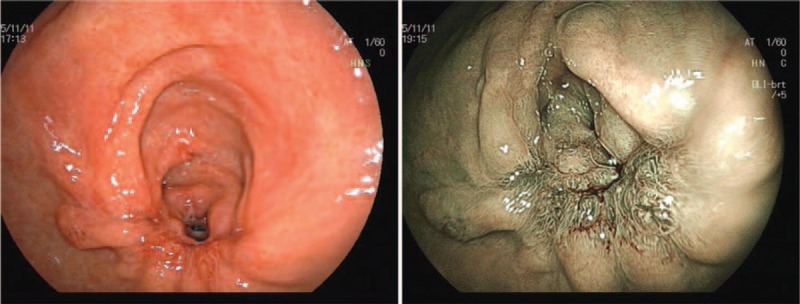
A gastroscopy on the 40th day after endoscopic surgery showed a narrow lumen of the stomach caused by ulcer healing.

A 53-year-old man was admitted to the local hospital with left upper abdominal pain lasting 2 weeks. A gastroscopy showed a bulging gastric lesion in the antrum. However, the pathology was benign. The patient did not receive any treatment, and still had abdominal pain. Then, he was referred to the Second Affiliated Hospital of Xi’an Jiaotong University. Physical examination revealed no mucocutaneous pigmentation, no palpable masses in the abdomen, but left upper abdominal tenderness. The patient had no family history of polyps or tumors in the gastrointestinal tract. Laboratory tests were normal, and the tumor markers were within normal limits. Abdominal computed tomography revealed that the gastric wall in the antrum was thickened (Fig. 1). A gastroscopy showed a giant and extensive bulging gastric lesion located on the greater curvature and posterior and anterior walls of the gastric antrum, involving three-quarters of the gastric wall. The mucosal surface of the lesion was smooth without erosion or ulcer. Magnifying endoscopy with narrow-band imaging showed the almost normal microvascular morphology of the lesion (Fig. 2). Endoscopic ultrasonography was performed, showing a low-echo area in the muscularis mucosae and no invasion into the submucosal layer, without enlarged celiac lymph nodes (Fig. 3). An endoscopic mucosal resection was performed, and a bigger specimen was sent for histopathological examination to confirm the diagnosis. The final pathology report indicated a hamartomatous polyp (Fig. 4). After excluding PJS, a solitary Peutz–Jeghers-type hamartomatous polyp was diagnosed. Then, the patient underwent an ESD. The resected specimen was measured in 110 × 80 × 4 mm^3^ (Fig. 5). We treated patients with acid suppression. The patient recovered quickly, without any complications. A gastroscopy on the fourth day after endoscopic surgery showed a large postoperative artificial ulcer (Fig. 6). A gastroscopy on the 40th day after endoscopic surgery showed a narrow lumen of the stomach caused by ulcer healing. Also, a polyp of 4 × 4 mm^2^ was observed in the lesser curvature of the gastric body (Fig. 7). The patient was followed up regularly.

## 3 Discussion

Gastrointestinal polyps are divided into hyperplastic, inflammatory, adenomatous, and hamartomatous polyps. Hamartomatous polyps are extremely rare and mainly occur in PJS.^[1]^ They are also found in Cronkhite–Canada syndrome, Cowden syndrome, and so on,^[2]^ which are extremely rare.

Peutz^[3]^ and Jeghers et al^[4]^ first described PJS. It is a rare autosomal dominant genetic disease characterized by mucocutaneous pigmentation (mouth, lips, hands, and feet) and familial gastrointestinal polyposis. Later, some hamartomatous polyps, confirmed by pathology without mucocutaneous pigmentation or a family history of digestive tract diseases, were reported. Since they lack the characteristics of PJS, they are known as solitary Peutz–Jeghers-type polyps.^[5]^

The incidence of solitary Peutz–Jeghers-type polyps is extremely low. The solitary Peutz–Jeghers-type polyp can be found in the gastrointestinal tract; more cases occur in the small intestine, followed by colorectal region. The solitary gastric Peutz–Jeghers-type polyps are the rarest.^[6]^ Kuwano et al^[7]^ reported a case of solitary gastric hamartomatous polyp in 1989 for the first time. So far, only 9 cases of the solitary gastric Peutz–Jeghers-type polyp were reported abroad (Table 1), and a few cases were reported in China, some of which were not adequately detailed.

**Table 1:**
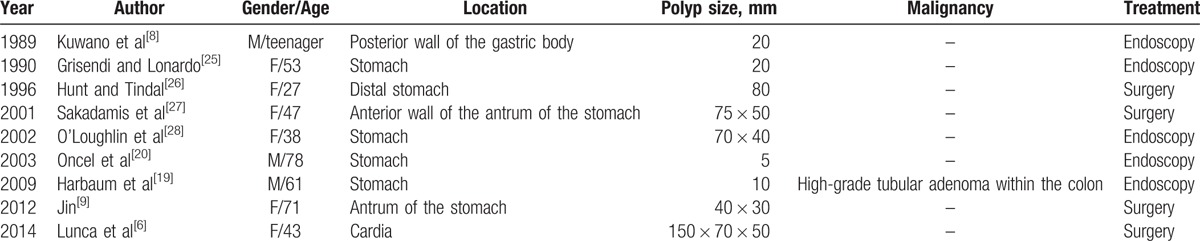
Previously reported cases with solitary gastric Peutz–Jeghers-type polyp.

The solitary Peutz–Jeghers-type polyps are often detected because of abdominal discomfort, abdominal pain, and nonspecific symptoms. Some patients are asymptomatic and discover the lesion accidentally during endoscopic examination. Some cases are found with intussusception, bleeding, pancreatitis, and so forth.^[8]^ A few cases are similar to the tumor, even with similar performance of tumor metastasis such as enlarged lymph nodes.^[6,9]^ Therefore, the doctor must be very careful during diagnosis. The diagnosis of solitary Peutz–Jeghers-type polyps should be based on pathology. The pathological feature of solitary Peutz–Jeghers-type polyps is that the smooth muscle bundles from the muscularis mucosae extend to the polyp and form a typical branch-like structure covered by almost normal mucosa. This is the same as PJS.^[10]^ The size of gastric Peutz–Jeghers-type polyps ranges from 5 to 80 mm in English reports. The largest gastric Peutz–Jeghers-type polyp, reported by Lunca, is 150 × 70 × 50 mm^3^ in size.^[6]^ The solitary hamartomatous polyp reported in this study was located in gastric antrum, involving three-quarters of the gastric wall. The postoperative specimen was measured in 110 × 80 × 4 mm^3^. It was the second largest gastric Peutz–Jeghers-type polyp. Lesions with large size and wide range are extremely rare.

No clear guidelines are available for the treatment of solitary Peutz–Jeghers-type polyps. Therefore, they are mainly treated by surgical resection or endoscopic resection. The endoscopic ultrasonography examination can help estimate the origin and blood supply of the lesion and its relationship with the surrounding tissues and organs. Hence, it has great significance in the diagnosis and choice of therapeutic method. In this study, the endoscopic ultrasonography showed the lesion limited to the muscularis mucosae and no invasion into the submucosal layer, without enlarged celiac lymph nodes. Therefore, ESD was adopted. Endoscopic treatment has many advantages such as mild trauma and rapid recovery. It is widely used in the treatment of gastrointestinal tract disease. The ESD technique has developed rapidly in recent years. It can resect larger lesions completely. The present case was also the largest gastric Peutz–Jeghers-type polyp treated by endoscopy.

Although more cases have been reported, it is still uncertain whether solitary Peutz–Jeghers-type polyp is an incomplete form of PJS or an entity different from PJS.^[11,12]^ No mucocutaneous pigmentation or a family history of PJS in older patients and low risk of tumor development without a malignant change in other organs may be the reasons why a solitary Peutz–Jeghers-type polyp is considered to be a clinical entity different from PJS.^[13,14]^ Hemminki et al^[15]^ identified the gene of PJS (STK11/LKB1) in 1997. The gene mutation occurs in more than 70% to 80% of the PJS.^[16,17]^ Only three cases were used to explore whether STK11 gene mutations occurred in solitary Peutz–Jeghers-type polyps. These three cases of solitary Peutz–Jeghers-type polyps occurred in the duodenum,^[18]^ stomach,^[19]^ and ileum,^[12]^ but the genetic analysis in all three cases did not reveal STK11 gene mutation. Therefore, the author considered that the solitary Peutz–Jeghers-type polyp was a clinical entity different from PJS.

PJS increases the risk of cancers of the digestive tract and other organs significantly, but the cancer risk of solitary Peutz–Jeghers-type polyp is still inconclusive. Oncel et al^[20]^ followed up 8 patients with solitary Peutz–Jeghers-type polyps for 11.5 years and found no hamartomatous polyp recurrence or tumor occurrence. Therefore, the hamartomatous polyp was considered as a benign lesion, which did not increase the risk of tumorigenesis. However, Burkart et al^[21]^ reviewed all reported cases of solitary Peutz–Jeghers-type polyps in the hospital in 22 years and concluded that solitary Peutz–Jeghers-type polyps are extremely rare, they are also associated with the risk of cancer similar to PJS, and they may be incomplete PJS. The literature in PubMed was searched using the key words “solitary,” “hamartomatous polyps,” “adenoma,” and “adenocarcinoma.” Eight cases with malignant components were found in the polyp or other organs (Table 2). Aneiros et al^[22]^ reported a solitary Peutz–Jeghers-type polyp with neoplastic transformation in the jejunum in 1988 for the first time. Harbaum at el^[19]^ reported a case of solitary Peutz–Jeghers-type polyp with intraepithelial neoplasia in the colon. This supported the hypothesis that both solitary Peutz–Jeghers-type polyp and PJS are associated with the risk of cancer. Arima^[23]^ reported a case of solitary Peutz–Jeghers-type polyp with the hamartoma–adenoma–carcinoma sequence. Limaiem et al^[24]^ reported a case of solitary rectal Peutz–Jeghers-type polyp with adenomatous transformation. Suzuki reviewed 19 cases of solitary duodenal Peutz–Jeghers-type polyp; 4 cases showed malignant transformation,^[13]^ and 1 of them had duplicated malignancy in 6 organs.^[14]^ Whether these case reports can explain the risk of cancer associated with Peutz–Jeghers-type polyps is still controversial Table 3.

**Table 2:**
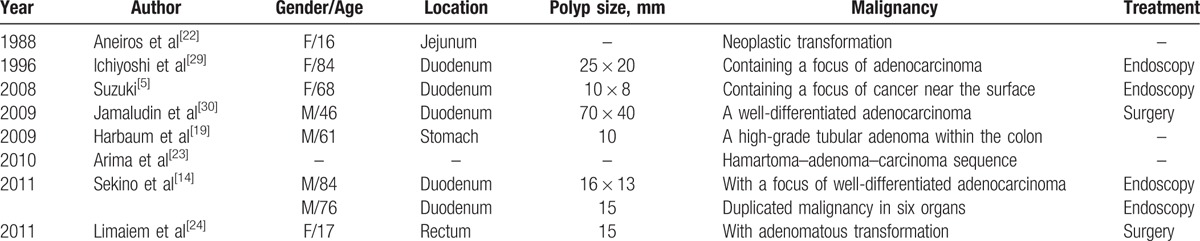
Solitary Peutz–Jeghers-type hamartomatous polyp with malignant transformation.

**Table 3:**
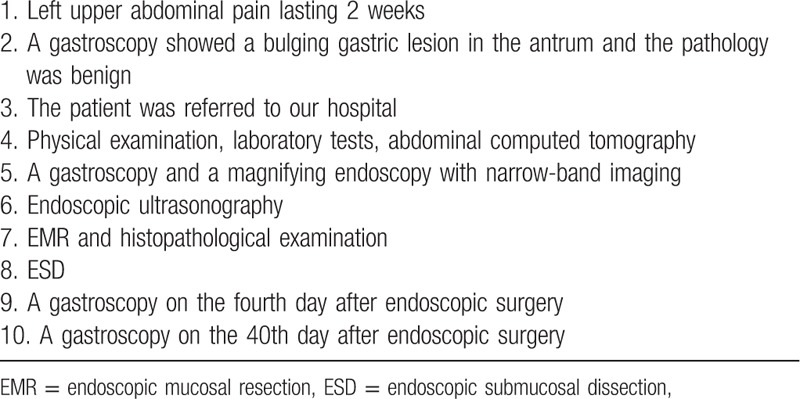
Timeline.

The case we reported is the second largest gastric solitary Peutz–Jeghers-polyp reported until now, and the largest gastric solitary Peutz–Jeghers type-polyp treated by endoscope. And the follow-up of these patients is necessary because of the possible risk of malignant transformation. A long-term follow-up can provide significant information to better understand this disease.
